# Evaluation of Errors Associated with Cutting-Induced Plasticity in Residual Stress Measurements Using the Contour Method

**DOI:** 10.1007/s11340-017-0255-5

**Published:** 2017-02-27

**Authors:** Y. L. Sun, M. J. Roy, A. N. Vasileiou, M. C. Smith, J. A. Francis, F. Hosseinzadeh

**Affiliations:** 1 School of Mechanical, Aerospace and Civil Engineering, The University of Manchester, Sackville Street, Manchester, M13 9PL UK; 2 Engineering and Innovation Department, The Open University, Walton Hall, Milton Keynes, MK7 6AA UK

**Keywords:** EDM cutting, Finite element analysis, Stress redistribution, Uncertainty, Welding

## Abstract

Cutting-induced plasticity can lead to elevated uncertainties in residual stress measurements made by the contour method. In this study plasticity-induced stress errors are numerically evaluated for a benchmark edge-welded beam to understand the underlying mechanism. Welding and cutting are sequentially simulated by finite element models which have been validated by previous experimental results. It is found that a cutting direction normal to the symmetry plane of the residual stress distribution can lead to a substantially asymmetrical back-calculated stress distribution, owing to cutting-induced plasticity. In general, the stresses at sample edges are most susceptible to error, particularly when the sample is restrained during cutting. Inadequate clamping (far from the plane of cut) can lead to highly concentrated plastic deformation in local regions, and consequently the back-calculated stresses have exceptionally high values and gradients at these locations. Furthermore, the overall stress distribution is skewed towards the end-of-cut side. Adequate clamping (close to the plane of cut) minimises errors in back-calculated stress which becomes insensitive to the cutting direction. For minimal constraint (i.e. solely preventing rigid body motion), the plastic deformation is relatively smoothly distributed, and an optimal cutting direction (i.e. cutting from the base material towards the weld region in a direction that falls within the residual stress symmetry plane) is identified by evaluating the magnitude of stress errors. These findings suggest that cutting process information is important for the evaluation of potential plasticity-induced errors in contour method results, and that the cutting direction and clamping strategy can be optimised with an understanding of their effects on plasticity and hence the back-calculated stresses.

## Introduction

Residual stresses are self-equilibrating stresses in a stationary material or structure which is free of external loads. They can be either detrimental or beneficial, depending on their role in interacting with in-service loading [1, 2]. From a design perspective, it is important to quantify the residual stresses developed via the manufacturing of engineering components as the residual stresses can be a key factor influencing damage and failure. A variety of techniques have been developed to measure residual stresses in metallic components [1, 3], among which the contour method [4] is relatively new. In comparison with some of the more commonly used techniques (e.g. X-ray/neutron diffraction and hole drilling), the contour method has several advantages, such as insensitivity to microstructural variation (grain/texture distribution), the capability of mapping stresses across an entire cross-section, and accessibility using relatively available machine tools and metrology equipment.

The contour method is a destructive strain release technique based on Bueckner’s principle of elastic superposition [5]. The standard contour method is implemented in the following steps [4, 5]: (1) experimentally cutting the sample, typically with wire electrical discharge machining (WEDM); (2) measuring the out-of-plane displacements (i.e. contour) of the created cut surfaces; (3) processing the measured data; and (4) back-calculating the residual stresses using finite element (FE) analysis for the majority of cases. In the final step of the contour method, normally, a 3D finite element model (FEM) of one of the cut parts is created and the negative values of the out-of-plane displacements (after data processing) are applied on the cut surface as nodal boundary conditions. A linear elastic stress analysis is then performed to reconstruct the residual stresses. Alternatively, Kartal [6] has developed analytical solutions for samples with simple geometry, which have been used to analyse residual stresses developed during welding [7]. Further details of the contour method are described elsewhere [5, 8, 9].

Cutting is the first and most critical step in the contour method. Several sources of errors in the cutting step could lead to significant uncertainties in the measured results. These include cutting artefacts, bulge errors and plastic deformation [5, 8, 10]. The errors (e.g. cutting artefacts), which are entirely associated with the nature of WEDM cutting and are independent of the stress state in the sample, can be avoided in most cases [8, 10]. Also, they could be rectified in the data processing step, or they could be corrected for by performing a test cut [4, 5]. The stress-dependent error sources, such as bulge and plasticity, are difficult to avoid or control. A bulge error occurs when the cut width is not constant with respect to the original configuration of the sample being cut and it arises from the change of the stress state (relative to the original stress state) at the cutting front as cutting progresses. Nevertheless, this error can be minimised by improved clamping, employing a thinner wire, or by carrying out a correction using an iterative FE analysis [5, 11].

In contrast, cutting-induced plasticity is more difficult to control and it cannot be corrected for after the sample is sectioned, and hence a good understanding of the effect and its mechanism is required. Plastic deformation is most common when the residual stress is close to the yield strength of a ductile material being cut. However, the inevitable localised stress concentration at the cutting front can lead to significant plastic deformation even when the far-field residual stress is less than the yield strength. The plastic deformation depends on the residual stress and its evolution during cutting, and consequently plasticity is challenging to characterise in experiments, resulting in elevated measurement uncertainties.

As cutting-induced plasticity violates the elastic stress-relaxation assumption on which the contour method is based, it is important to evaluate its effect on the back-calculated stresses. Experimental evaluation has been attempted by comparison of the contour method with other stress measurement techniques [12–14]. However, there are other sources of error than plasticity that could contribute to disagreements (if they exist) between contour method stress measurements and other stress measurements, and thus it may be difficult to isolate the contribution of cutting-induced plasticity to the observed errors (if any) in stress. Moreover, other stress measurement techniques (e.g. X-ray/neutron diffraction and hole drilling) have their own sources of errors [1, 3], generating uncertainties in the residual stresses measured with these techniques, and this is significant because such measurements are often used for error evaluation of contour method measurements. To overcome the limitations of experimental evaluation, some numerical studies have been carried out. For instance, 2D cutting simulations based on a given idealised stress distribution have been used previously to gain insight into the effects of constraint strategy on errors in stress [15, 16], but they seemed over simplified and incapable of capturing the effects of complicated 3D stresses and deformation during cutting. Dennis et al. [17] conducted 3D FE modelling of contour cutting for ring and groove welds, in which realistic weld residual stresses were generated using welding models, but the effects of different cutting directions on the resulting plasticity errors were not explored. Traore et al. [14] proposed a novel cutting approach employing sample self-constraint, based on a 2D elastic fracture mechanics analysis, to minimise the stress intensity factor during cutting and hence the risk of plasticity-induced errors. However, the claimed benefits of this approach need to be rationalised using a 3D plastic analysis model. Recently, Hosseinzadeh et al. [18] extended the self-constraint approach by employing a double-embedded cutting configuration, thereby improving the stress measurement accuracy in comparison with the conventional contour method. Muránsky et al. [19] provided 3D cutting simulation results to understand the mechanism of plasticity mitigation in the novel approach developed by Hosseinzadeh et al. [18]. These previous studies focussed on either idealised residual stress distributions or specific cutting directions, and they proved to be highly valuable.

In this study, cutting-induced plasticity and its implications for the accuracy of stress measurements made using the contour method are numerically investigated, aiming to understand the plasticity-controlled mechanism for generating errors in back-calculated stresses. This is done through employing a benchmark edge-welded beam having a realistic residual stress distribution and suitable complexity. First, the residual stresses in the beam are determined using a validated, sequentially coupled thermo-mechanical welding model. Then cutting simulations are performed using different cutting configurations (i.e. different cutting directions and clamping strategies). Experimental results obtained using the contour method [20] are also included for comparison. The effects of cutting-induced plasticity on the back-calculated stresses are analysed and discussed.

## Description of Problem and Method

### Benchmark Edge-Welded Beam and Weld Modelling

**Fig. 1:**
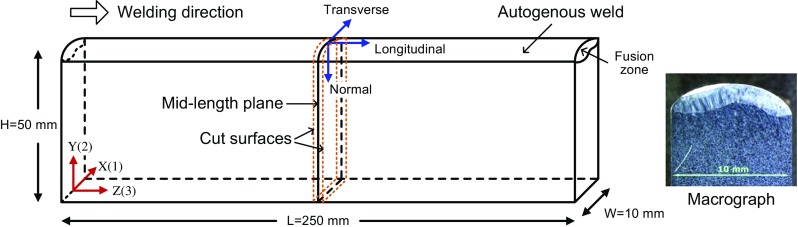
Benchmark sample comprising an AISI grade 316H stainless steel beam for which one edge was welded autogenously using the tungsten inert gas (TIG) technique. One transverse weld macrograph is also included. Further details are available in Ref. [21]. Contour cutting is simulated through the mid-length plane

Edge-welded beams have been produced as benchmarks to guide weld modelling practice [21]. An autogenously TIG-welded beam sample made of austenitic AISI 316H steel [21] was used for the contour cutting simulation in this study, as shown in Fig. 1. This benchmark was selected as the subject owing to its simple geometry, as well as for the fact that no filler material was added during its manufacture, and due to the absence of complicated solid-state phase transformations over the majority of the sample. Comprehensive information, e.g. welding parameters, material properties and processed experimental data, are available in Refs. [21, 22] for model validation. This benchmark is an ideal test case (a) for predicting weld residual stresses by FE modelling with sufficient accuracy, (b) for validating a weld FE model using the reported experimental data [20, 21] and (c) for using the validated FE model to simulate the entire contour method process.

First, weld modelling was carried out, consisting of three steps: (1) calibration of a 3D moving heat source; (2) thermal analysis; and (3) mechanical analysis. In the first step, a numerical tool (FEAT-WMT) was used to derive an ellipsoidal heat source involving lateral motion (weaving) with respect to the centre line of the specified weld region [23]. The ellipsoidal heat source is defined by

1 $$ q=\frac{Q}{V_a} \exp \left(-\left[{\left(\frac{x-{x}_{\mathrm{c}}}{r_l}\right)}^2+{\left(\frac{y-{y}_{\mathrm{c}}}{r_v}\right)}^2+{\left(\frac{z-{z}_{\mathrm{c}}}{r_a}\right)}^2\right]\right) $$

where *q* is the power per unit volume, *Q* is the total input power, (*x*_*c*_, *y*_*c*_, z_*c*_) is the geometric centre of the ellipsoidal heat source, and *r*_*l*_, *r*_*v*_ and *r*_*a*_ are the radii of the distribution in the lateral (*x*), vertical (*y*) and axial (*z*) directions, respectively. The quantity *V*_*a*_ is calculated by using the following integral so that the total input power is always equal to *Q* even when the source moves.

2 $$ {V}_a={\int}_{V_{\mathrm{work}}} \exp \left(-\left[{\left(\frac{x-{x}_{\mathrm{c}}}{r_l}\right)}^2+{\left(\frac{y-{y}_{\mathrm{c}}}{r_v}\right)}^2+{\left(\frac{z-{z}_{\mathrm{c}}}{r_a}\right)}^2\right]\right)\ \mathrm{d} V $$

where *V*_*work*_ is the total volume of materials considered. During calibration, the heat source was moved along the axial (*z*) direction and was also oscillated laterally to capture the effect of the torch weaving during the experiments.

The calibrated heat source was then input into the general purpose FE code Abaqus to predict the welding temperature history in the beam via a thermal analysis. In a subsequent mechanical analysis the transient temperature fields were imposed on the same mesh (Fig. 5(a)) that was used in the thermal analysis, and a combined hardening plasticity model (Lemaitre-Chaboche) was employed to account for plastic material behaviour [24, 25]. Combined hardening models have been shown to be best suited to describing the cyclic plastic behaviour of austenitic Type 316 stainless steel [25, 26]. It has been also demonstrated that the use of combined hardening models and moving heat sources can most accurately capture the residual stresses in stainless steel welds [22, 23, 25, 27–29].

**Fig. 2:**
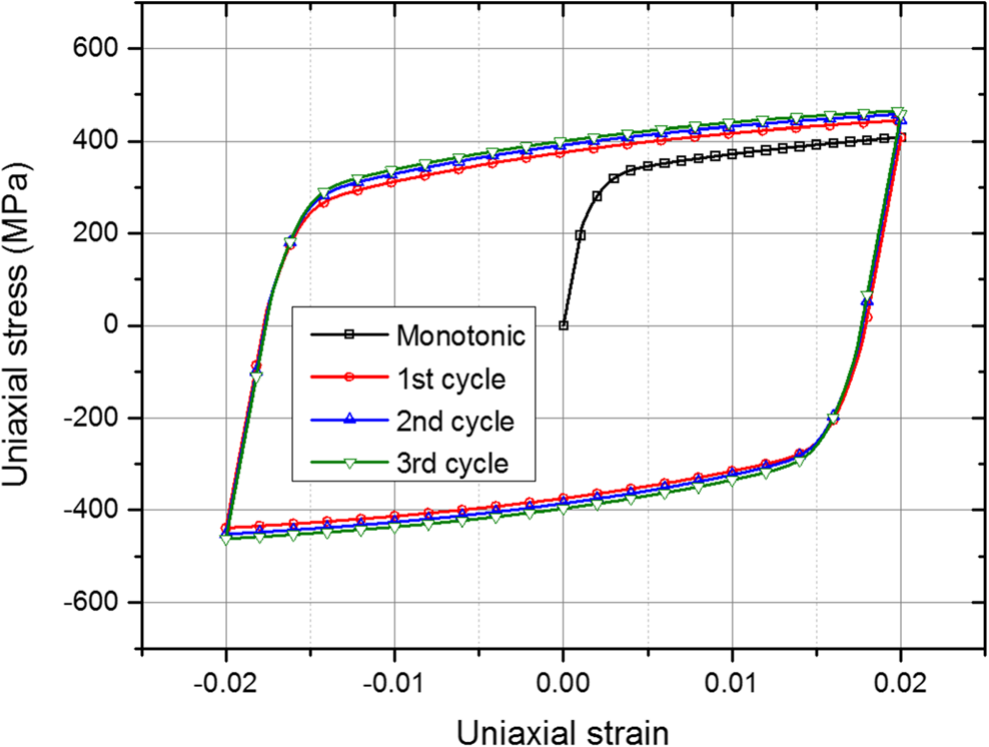
Uniaxial cyclic stress-strain curves predicted by the calibrated combined hardening model with room-temperature material parameters as provided in Table 1

In the combined hardening model, the kinematic hardening components or backstresses are superposed, and the hardening law for each backstress is

3 $$ {\dot{\boldsymbol{\upalpha}}}_k={C}_k\frac{1}{\sigma^0}\left(\boldsymbol{\upsigma} -\boldsymbol{\upalpha} \right){\dot{\overline{\varepsilon}}}^{\mathrm{pl}}-{\gamma}_k{\boldsymbol{\upalpha}}_k{\dot{\overline{\varepsilon}}}^{\mathrm{pl}} $$

and the overall backstress is computed by

4 $$ \boldsymbol{\upalpha} =\sum_{k=1}^N{\boldsymbol{\upalpha}}_k $$

where ${\dot{\overline{\varepsilon}}}^{\mathrm{pl}}$ is the equivalent plastic strain rate, *σ* ^0^ is the size of the yield surface, and *C*_*k*_ and γ_*k*_ are material parameters. The isotropic hardening is defined by using the following exponential law

5 $$ {\sigma}^0={\left.\sigma \right|}_0+{Q}_{\infty}\left(1-{e}^{- b{\overline{\varepsilon}}^{\mathrm{pl}}}\right) $$

where *σ*|_0_ is the yield stress at zero plastic strain, and *Q*_∞_ and *b* are material parameters. The temperature-dependent Lemaitre-Chaboche parameters for the beam material (AISI 316H steel) were obtained by Aird et al. [22, 27] using appropriate monotonic and cyclic test data, which are also listed in Table 1. Details relating to the combined hardening parameters for stainless steels and their determination are described in Ref. [25]. The uniaxial cyclic stress-strain curves at room temperature, as predicted by the combined hardening model, are shown in Fig. 2. An annealing temperature of 1400 °C was assumed in the weld model. For the cutting simulation, only the room-temperature material parameters were used.

**Table 1:** Lemaitre-Chaboche combined kinematic-isotropic hardening parameters [22, 27]

Temperature (°C)	*σ*|_0_ (MPa)	*C*_1_ (MPa)	γ_1_	*C*_2_ (MPa)	γ_2_	*Q*_∞_ (MPa)	*b*
20	216.5	156,435.0	1410.85	6134.0	47.19	62.5	6.9
275	165.6	100,631.0	1410.85	5568.0	47.19	86.7	6.9
550	147.7	64,341.0	1410.85	5227.0	47.19	93.8	6.9
750	117.3	56,232.0	1410.85	4108.0	47.19	12.0	6.9
900	114.1	49,588.0	1410.85	292.1	47.19	0.0	6.9
1000	54.9	0.0	1410.85	0.0	47.19	0.0	6.9
1100	34.0	0.0	1410.85	0.0	47.19	0.0	6.9
1400	3.7	0.0	1410.85	0.0	47.19	0.0	6.9

**Fig. 3:**
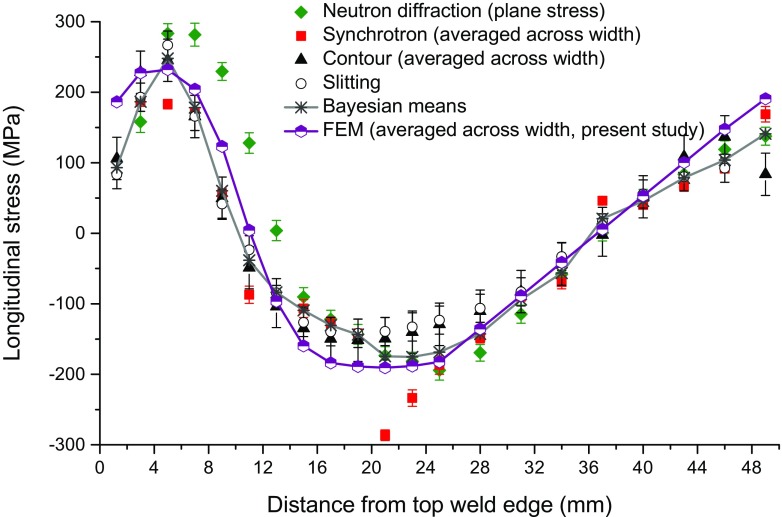
Comparison of the longitudinal residual stress distributions obtained by the present welding model and previous experimental measurements [21]

The thermal and mechanical predictions arising from the welding model meet all accuracy targets set in the benchmark document [21]. The root mean square error of the predicted rises in temperature at four thermocouple positions is 7%. The double lobe fusion boundary shape (see macrograph in Fig. 1) was captured and the predicted cross-sectional area of the fusion zone at the mid-length position of the beam is slightly (11%) smaller than the measured fused area. Figure 3 compares the predicted longitudinal stresses averaged across the width at the mid-length position of the beam with measured results obtained using different experimental techniques. Tensile stresses are produced both in the fusion zone and at the base of the sample, while compressive stresses cover the middle region. The root mean square error of the weld residual stresses predicted by the present welding model with respect to the Bayesian means of the experimental data is 39 MPa, approximately equal to 10% of the parent material (1%) proof stress at room temperature. In this case, the welding model appears to overestimate the residual stresses slightly, in comparison with the Bayesian means, as well as when compared to the contour method result, as shown in Fig. 3.

### Simulation of Contour Cutting

**Fig. 4:**
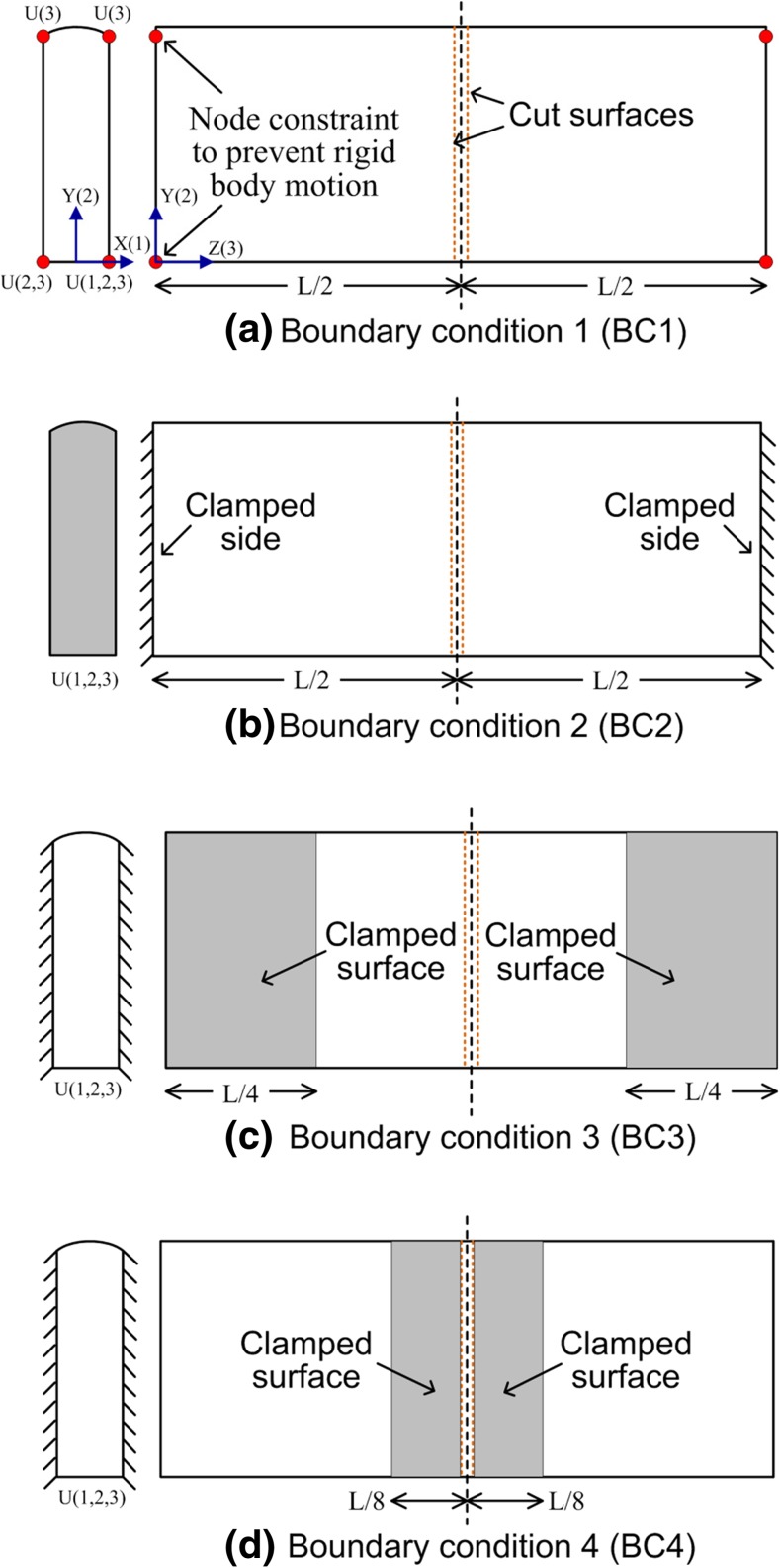
Illustration of typical boundary conditions adopted during contour cutting in the FE simulations. Note that BC1 represents a least-constraint approach, whereas BC2-BC4 are representative of typical clamping (idealised by completely fixing the FE nodes) during a contour cutting

The weld residual stresses predicted by the validated FE model were used to perform contour cutting simulations with different boundary conditions imposed, as summarised in Fig. 4. For boundary condition 1 (BC1), a least-constraint approach was applied to solely prevent rigid body motion during cutting. For boundary conditions 2 and 3 (BC2 and BC3), clamping is imposed either at the sample ends (lengthwise) for BC2 or on the sample surfaces near the sample ends (lengthwise) for BC3; these two clamping configurations are defined here as inadequate clamping since the clamping that is applied is relatively far from the plane of cut. Boundary condition 4 (BC4) represents clamping of the sample surface immediately adjacent to the plane of cut, and can be regarded as an example of adequate clamping. It should be noted that in the simulations the clamping is idealised and modelled by completely fixing the FE nodes at the clamping positions. Such an idealised scenario is unlikely to be achievable in practice due to material compliance. Nevertheless, the boundary conditions adopted here are deemed sufficient for the evaluation of the effects of typical clamping strategies on the results obtained with the contour method.

**Fig. 5:**
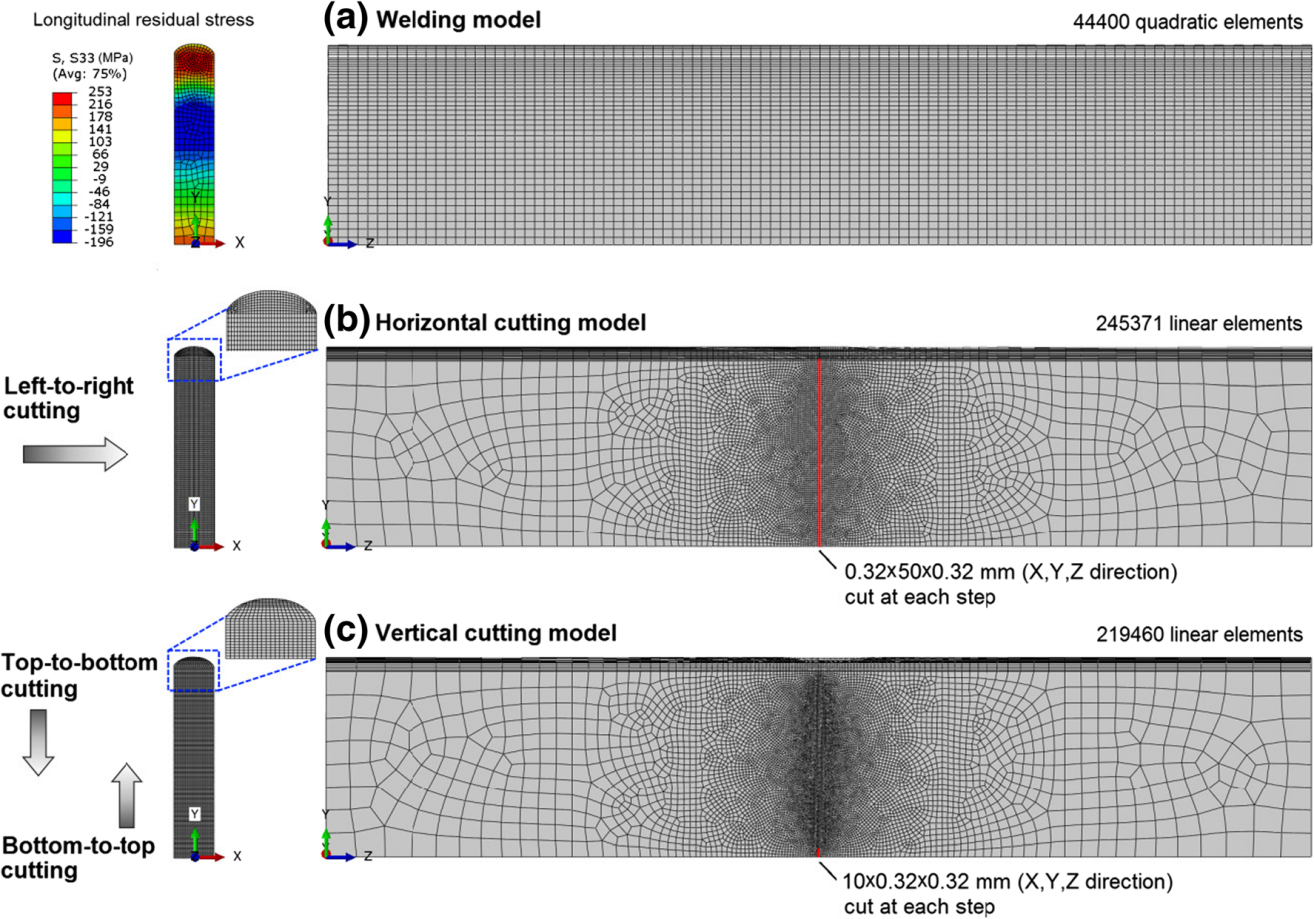
The FE meshes used in the welding and contour cutting models. The cutting path and the volume of material removed at each cutting step are also illustrated

Figure 5 shows the FE meshes used in the welding and cutting models, as well as the cutting paths and steps. Different meshes were employed for the welding and cutting simulations, consisting of quadratic and linear elements with reduced integration, respectively. The stress and strain results, along with other material variables required by the combined hardening plasticity model, were obtained from the weld modelling and mapped to the mesh that was used in the cutting simulation as initial condition. Note that weld distortion was ignored in the cutting model, which simplified the solution mapping procedure, and had little effect on the results, owing to the deformations that occurred during welding being small.

As the fusion zone and residual stresses are approximately symmetric about YZ plane (see Figs. 1 and 5(a)), only one cutting direction (left-to-right, Fig. 5(b)) was considered for the horizontal cutting model. In contrast, two cutting directions, i.e. top-to-bottom and bottom-to-top, were considered when the cutting was implemented vertically (Fig. 5(c)).

The contour cutting was simulated via the “model change” procedure in Abaqus/Standard [24]. A strip of material throughout the beam height (for horizontal cutting) or beam width (for vertical cutting) was ‘cut’ by removing the elements in the mid-length plane at each cutting step. The strip was 0.32 mm thick in both longitudinal and cutting directions in order to represent a typical cut made using a wire with a diameter of 0.25 mm in WEDM. A stress rebalance was performed in the beam after each increment of material removal. The cutting continued until the whole slice of material, originally located in the mid-length plane, was removed. Such discretization of the cutting process is justified by the fact that the contour cutting is usually conducted in a slow and stable manner, and because the thickness of material that is removed is comparable to a single wire diameter in WEDM.

### Determination of Surface Displacement and Residual Stress

For the experiment conducted on an identical benchmark sample, as described in Ref. [20], the beam was clamped solely on one side so as to implement both slitting and contour method measurements in tandem. A coordinate measuring machine was employed to measure the surface displacements (i.e. the out-of-plane displacements on the cut surface), having a measurement accuracy of 4.9 μm and a point spacing of 0.5 × 0.5 mm. In the modelling, the surface displacements on completion of cutting were directly extracted from the out-of-plane nodal displacements, and the spacing of the nodes on the cut surface was 0.32 × 0.50 mm. Finally, the averaged displacements for the two cut surfaces were applied as nodal boundary conditions to one half of the same mesh in the cutting model to back-calculate the residual stresses using a linear elastic FE analysis. The surface displacements obtained from the FE cutting simulations do not contain any errors associated with WEDM setup or surface measurements or data fitting, which are required in an experiment, thereby permitting the direct evaluation of cutting-induced plasticity effects on the back-calculated residual stresses of the “numerically welded” beam sample.

## Results

### Surface Displacement

**Fig. 6:**
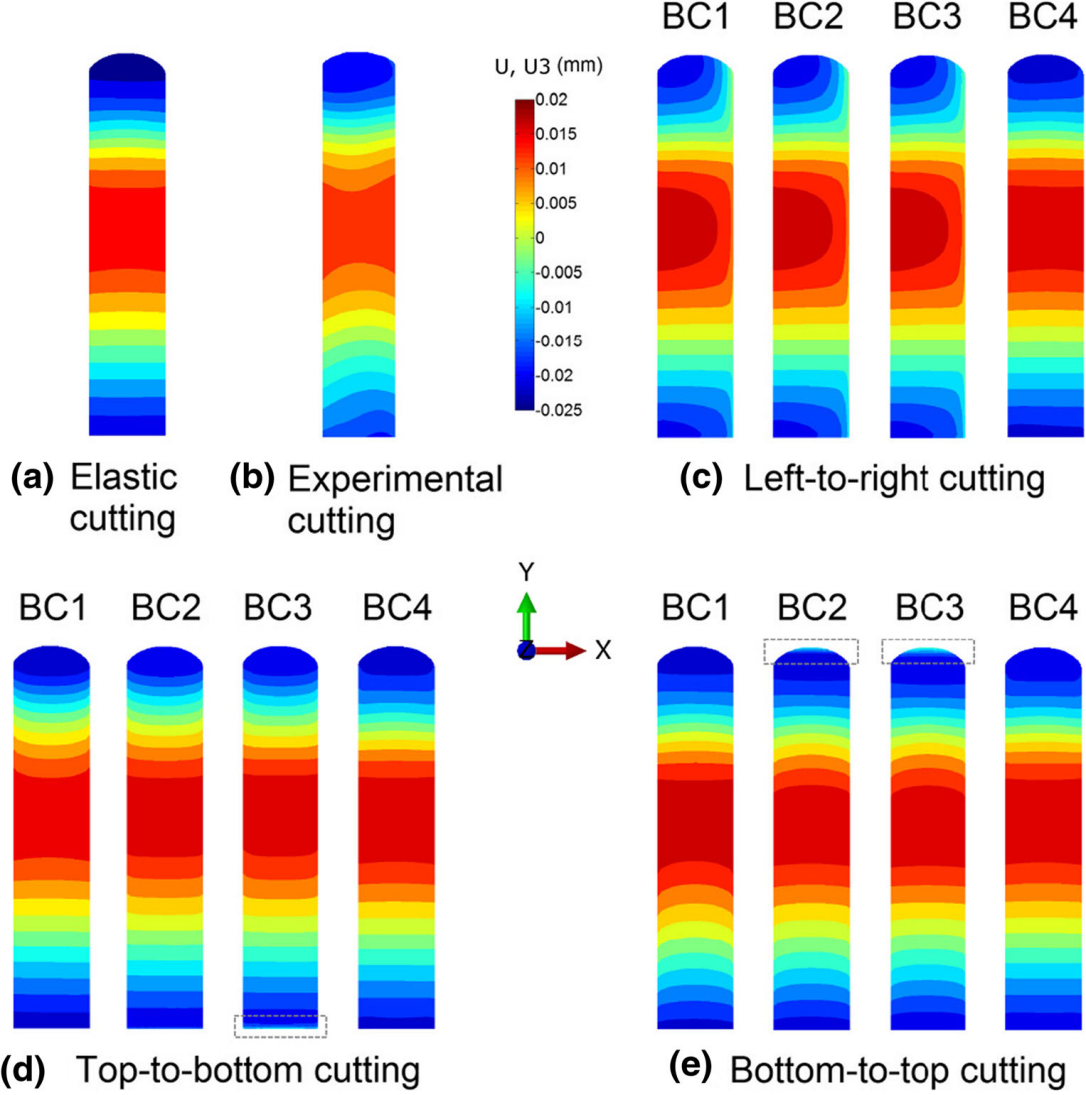
Contour maps showing averaged surface displacements for different cutting directions and boundary conditions. Note that in the elastic cutting simulation only linear elastic material properties are employed. The averaged surface displacement obtained from experiment [20] is also shown

Figure 6 shows the contour maps of the averaged surface displacements for different cutting strategies. The surface displacements extracted from elastic cutting (realised by mapping the weld residual stress to an elastic FE model and more information see “Simulation of Contour Cutting” section), where the material behaves elastically during cutting, are shown in Fig. 6(a). This absolutely elastic scenario may not be achievable in practice, but it provides a reference for comparison of the different numerical results. The experimental surface displacements obtained by top-to-bottom cutting [20] are included in Fig. 6(b), which, overall, exhibit similar features to the top-to-bottom cutting simulations, except for the discernible asymmetry, particularly in the bottom region. This asymmetry feature is attributed to the nature of the cutting required for the concurrent slitting measurement, i.e. the sample was clamped only on one side.

In the elastic case (Fig. 6(a)), the surface displacements are symmetric about the mid-width; this is consistent with the stress distribution after welding (see Fig. 5(a)). However, it is evident that horizontal cutting (i.e. left-to-right cutting shown in Fig. 6(c)) of an elasto-plastic material does not capture this symmetry. The magnitude of the resulting asymmetry is dependent on the boundary conditions and is minimised by adequate clamping (i.e. BC4) for which a similar contour to the elastic case is produced. For vertical cutting, symmetry about the mid-width plane is preserved, but the accuracy of the surface displacements in comparison with the ideal elastic case is also dependent on the boundary conditions. The overall accuracy generally increases from BC1 to BC4 as the efficacy of clamping increases. It is also noted that the most significant deviation from the elastic case appears in the upper part and lower part of the sample for the top-to-bottom cutting and bottom-to-top cutting, respectively, when BC1 is applied. Using the BC2 and BC3 clamping strategies, the results are overall brought closer to the elastic case, but these forms of inadequate clamping can introduce new errors at edges. For instance, when BC3 is applied, unexpected displacements are observed at the top edge for bottom-to-top cutting.

### Cutting-Induced Plasticity

**Fig. 7:**
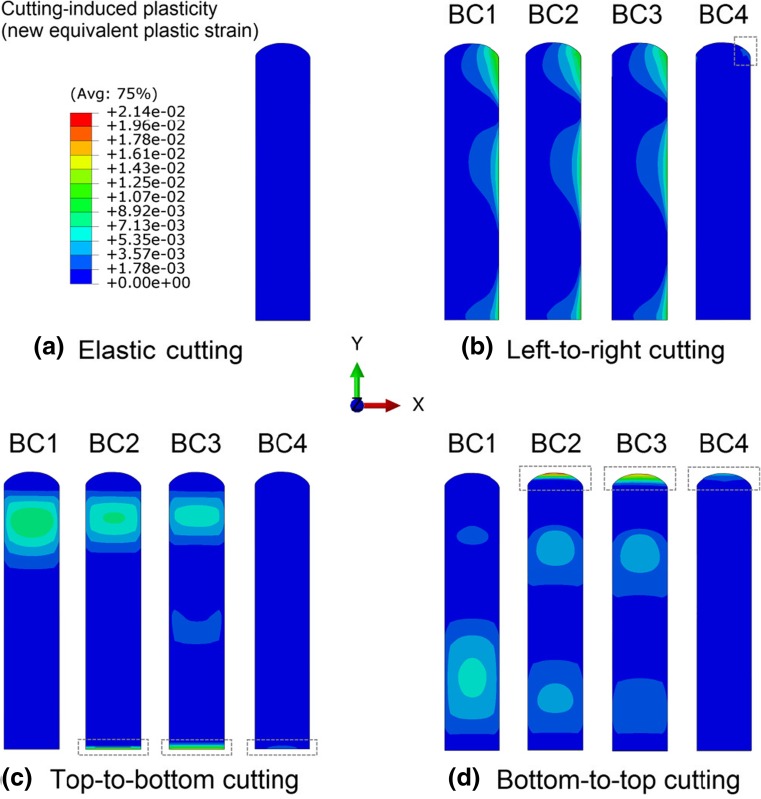
Cutting-induced plastic deformation (i.e. increase in equivalent plastic strain after cutting) on the cut surface for different cutting directions and boundary conditions. Note that in the elastic cutting simulation only linear elastic material properties are employed

Plastic deformation, which had accumulated after the welding simulation, further increased during the cutting simulation. Figure 7 shows contour maps of the cutting-induced plastic strain (i.e. the equivalent plastic strain due to the cutting process). It can be clearly seen that the cutting-induced plasticity depends on both the cutting direction and the boundary condition. An asymmetrical distribution of plastic strain is produced for left-to-right cutting, as shown in Fig. 7 (b), which explains the asymmetrical surface displacements for left-to-right cutting (Fig. 6(c)). The plastic strain distributions for top-to-bottom cutting and bottom-to-top cutting are also consistent with the corresponding surface displacement results (Fig. 6(d) and (e)), i.e. the highest deviation of the surface displacements from the elastic case occurs in the regions where plastic deformation is concentrated. Increasing the efficacy of the applied constraint, from BC1 to BC4, contributes to the overall mitigation of the cutting-induced plasticity. For all the cutting strategies considered, BC4 almost prevents the development of plastic deformation except at the edge of the sample (as highlighted by the dashed rectangles in Fig. 7). These highly localised plastic strains are produced where cutting ends. It is interesting to see that the plastic strain produced by left-to-right cutting is relatively insensitive to the boundary condition when changing from BC1 through to BC3, in contrast to the top-to-bottom and bottom-to-top cuts, in which the plastic strain distribution varies significantly when constraint increases.

**Fig. 8:**
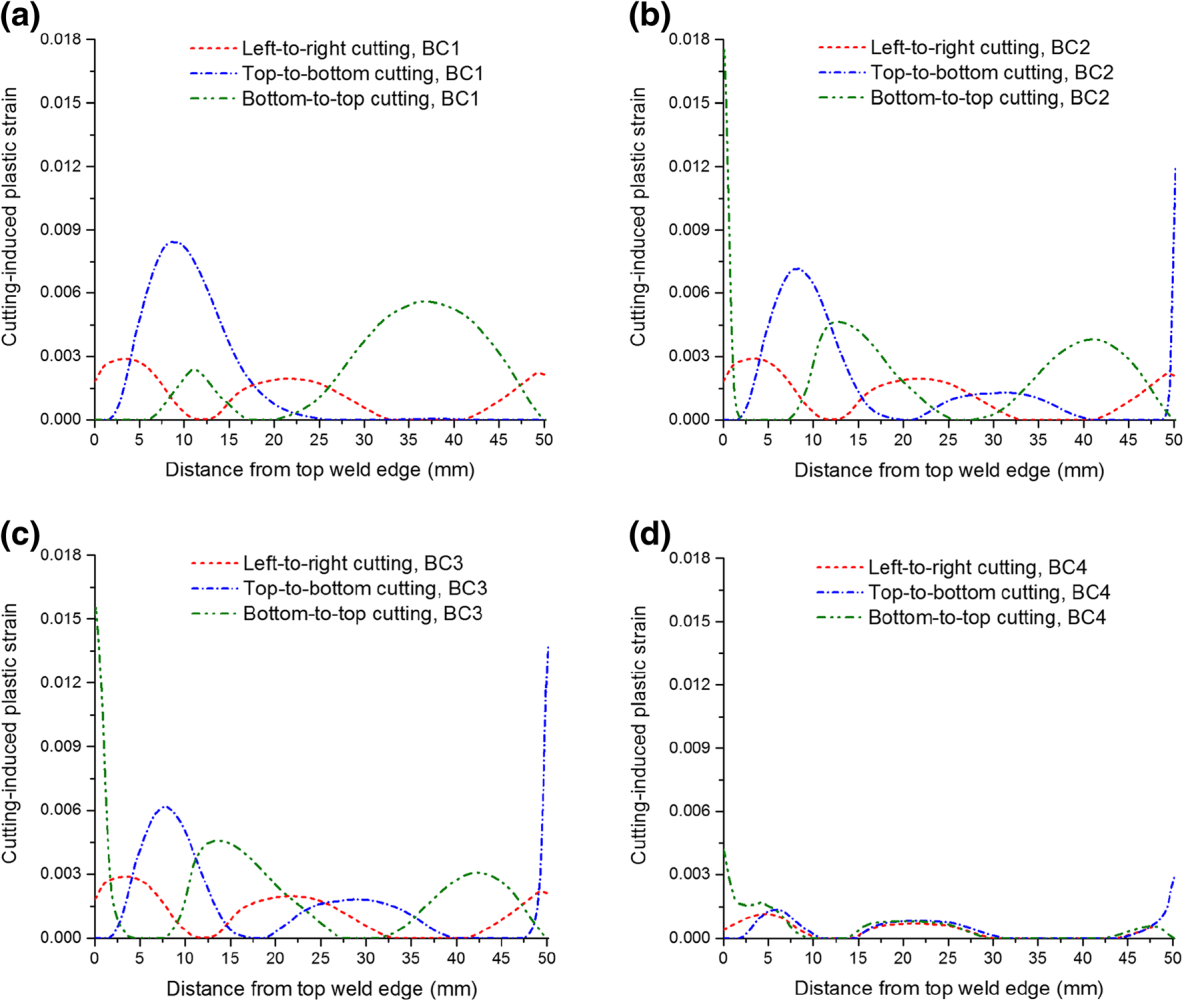
Line profile comparisons of the cutting-induced equivalent plastic strains at the mid-width position on the cut surface for different cutting strategies

Figure 8 plots the cutting-induced equivalent plastic strain along a line located at the mid-width position on the cut surface, for different cutting strategies, in order to highlight the effects of the cutting directions and boundary conditions. In the least-constraint case (BC1), most of the plastic deformation is smoothly distributed in the top part and the bottom part for the top-to-bottom and bottom-to-top cutting cases, respectively, i.e. in the high-stress region that was cut first. On the contrary, the cases with inadequate clamping (BC2 and BC3) lead to highly concentrated plastic deformation near the edges where cutting ends, i.e. the top edge and the bottom edge for bottom-to-top cutting and top-to-bottom cutting, respectively. The adequate clamping case (BC4) effectively mitigates the plasticity, but the plastic strain concentration on the end-of-cut site is still not completely eliminated. Left-to-right cutting produces relatively small plastic strains at the mid-width position, exhibiting insensitivity to the boundary condition. However, in such a case, the largest plastic strain is produced on the right-hand side of the beam sample, see Fig. 7 (b). For the three cutting directions, the bottom-to-top cutting with BC1 produces lowest plastic strains which, however, have widest spread, except BC4 of course.

### Residual Stress

**Fig. 9:**
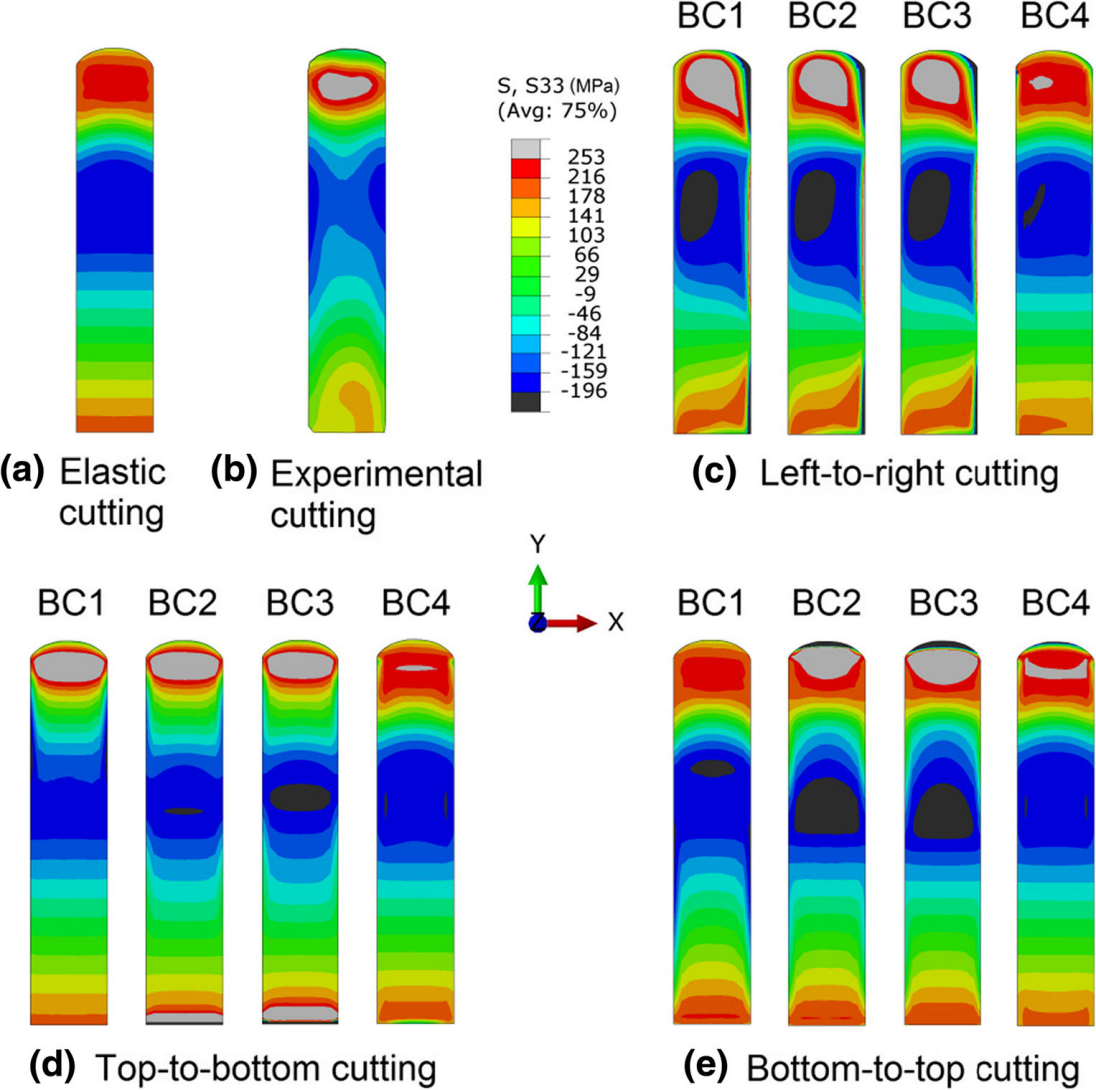
Back-calculated longitudinal residual stresses from the contour cutting simulation, and the corresponding stress results from the experiment [20]. Note that in the elastic cutting simulation only linear elastic material properties are employed

Figure 9 shows contour maps of the longitudinal stresses on the cut surface (see Fig. 1 for details of location) for different cutting strategies, along with experimental result [20]. The simulation captures the response of the beam sample to the cutting. For top-to-bottom cutting, the back-calculated stresses compared well between the simulation and experiment, see Fig. 9(b) and (d), as well as the associated surface displacements shown in Fig. 6(b) and (d).

The errors in the stresses determined using the contour method are evaluated here with respect to the elastic case, since the back-calculated stresses using the elastic model do not include any plasticity errors and are found same as the original residual stresses on the cut surface. When cutting-induced plasticity occurs, the reconstructed stress results have different degrees of errors. For left-to-right cutting (see Fig. 9(c)), the symmetry of the stresses about the mid-width plane (see Fig. 9(a)) is not captured. The errors are largest on the right-hand side where cutting ends, when any of BC1-BC3 are imposed. Adequate clamping, e.g. BC4, largely eliminates the artificial asymmetrical stress features. For top-to-bottom cutting (Fig. 9(d)) and bottom-to-top cutting (Fig. 9(e)), stress symmetry is captured, but the stress errors are still significant, unless BC4 is imposed. For most cases shown in Fig. 9, the stress distribution exhibits a skew trend towards the end of the cut, and the sample edges are most susceptible to errors in stress. It is also interesting to see that, for bottom-to-top cutting, the least-constraint condition (BC1) actually leads to better reconstructed stresses in terms of the magnitude of the errors, in comparison with the inadequate clamping cases (BC2 and BC3). This is because inadequate clamping reduces the overall plasticity but it introduces highly localized plasticity in the weld region, as shown in Fig. 7(d), which significantly increases the magnitude of the errors in stress in this region.

Recalling the maps of surface displacements (Fig. 6) and also noting the back-calculated stresses (Fig. 9) for different cutting strategies, it becomes clear that the resulting stresses are highly influenced by the boundary conditions that are used to represent the clamping condition during the cutting step. Thus the discrepancies between the numerical predictions (Fig. 9(d)) and experimental results (Fig. 9(b)) for the top-to-bottom cutting strategy can be mainly attributed to the fact that the boundary conditions used in the FE models, to simulate the clamping of the sample, do not perfectly represent the actual clamping strategy used in the experiment [20].

**Fig. 10:**
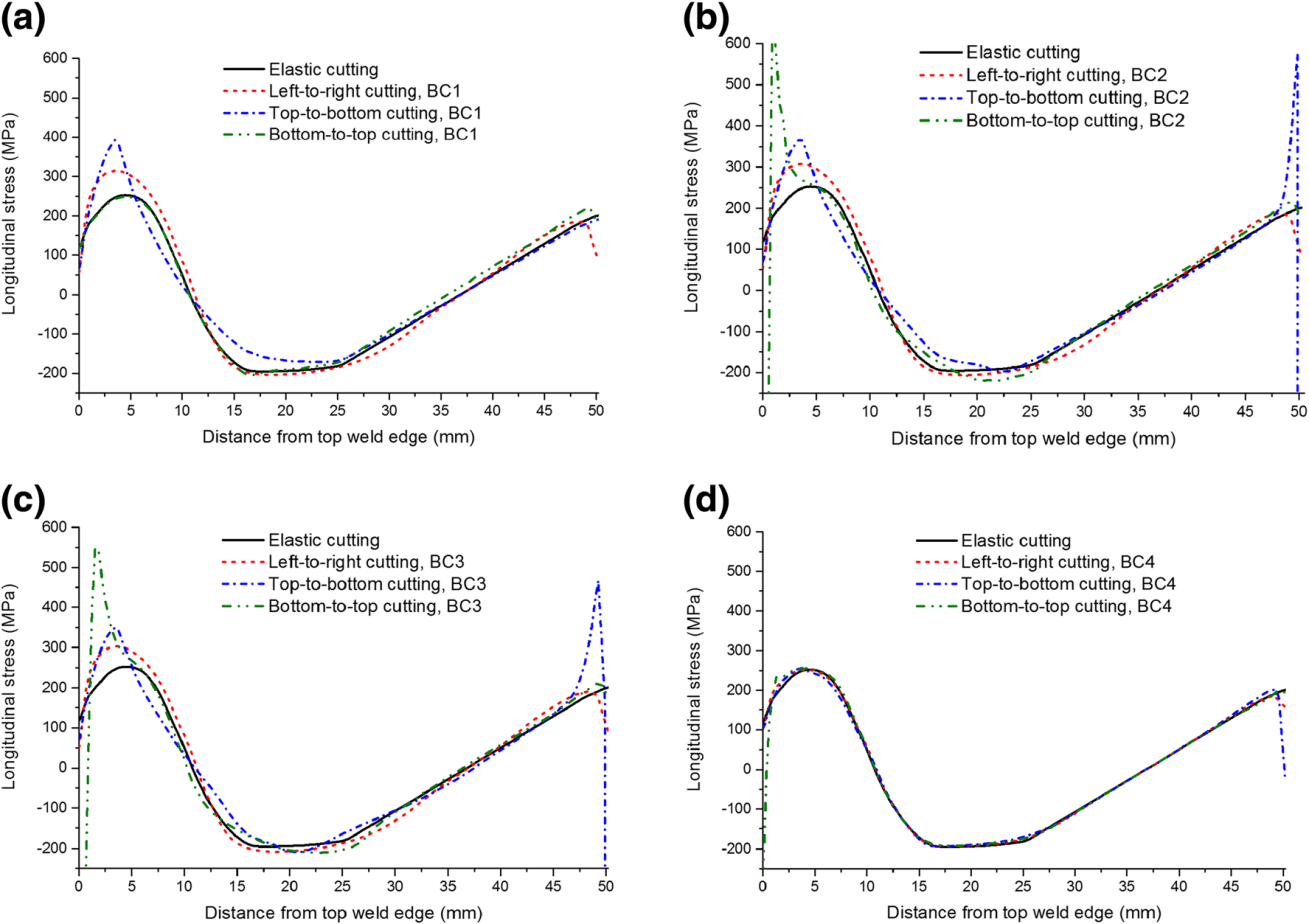
Back-calculated longitudinal residual stress distributions along a line profile at the sample mid-width position for different cutting strategies

Figure 10 shows the back-calculated longitudinal stresses along a line at the mid-width position of the beam. The plasticity-induced errors in stress are significant in some local regions, especially when insufficient constraint (i.e. cases BC1-BC3) is exerted. Recalling the cutting-induced plastic strain distribution (Fig. 8), it is found that the errors in stress are most significant in those regions where plastic deformation is high. However, it should be noted that the left-to-right cutting leads to essentially asymmetrical stress distributions with high errors in stress occurring on the right-hand side (as revealed in Fig. 9(c)), which is not reflected in the mid-width stress profile shown in Fig. 10. It appears that errors in stress are most likely to occur at the start-of-cut stage for the least-constraint case (BC1), but at the end-of-cut stage for clamping options BC2-BC4.

In comparison with BC1, the BC2 and BC3 conditions enhance the constraint during cutting and reduce the errors in stress in the regions where cutting starts and proceeds, but they lead to exceptionally high stress levels and gradients where cutting ends, due to the concentrated plastic strain there (see Figs. 7 and 8). Errors in stress can be minimised by employing adequate clamping (e.g. BC4), although considerable errors (e.g. at locations where the stress drops abruptly) still exist at the edge where cutting ends.

## Discussion

Simulation of the entire contour method process provides a useful means for evaluating the accuracy of contour results and the influence of cutting-induced plasticity, and to investigate the plasticity mechanism for different cutting directions and clamping arrangements.

**Fig. 11:**
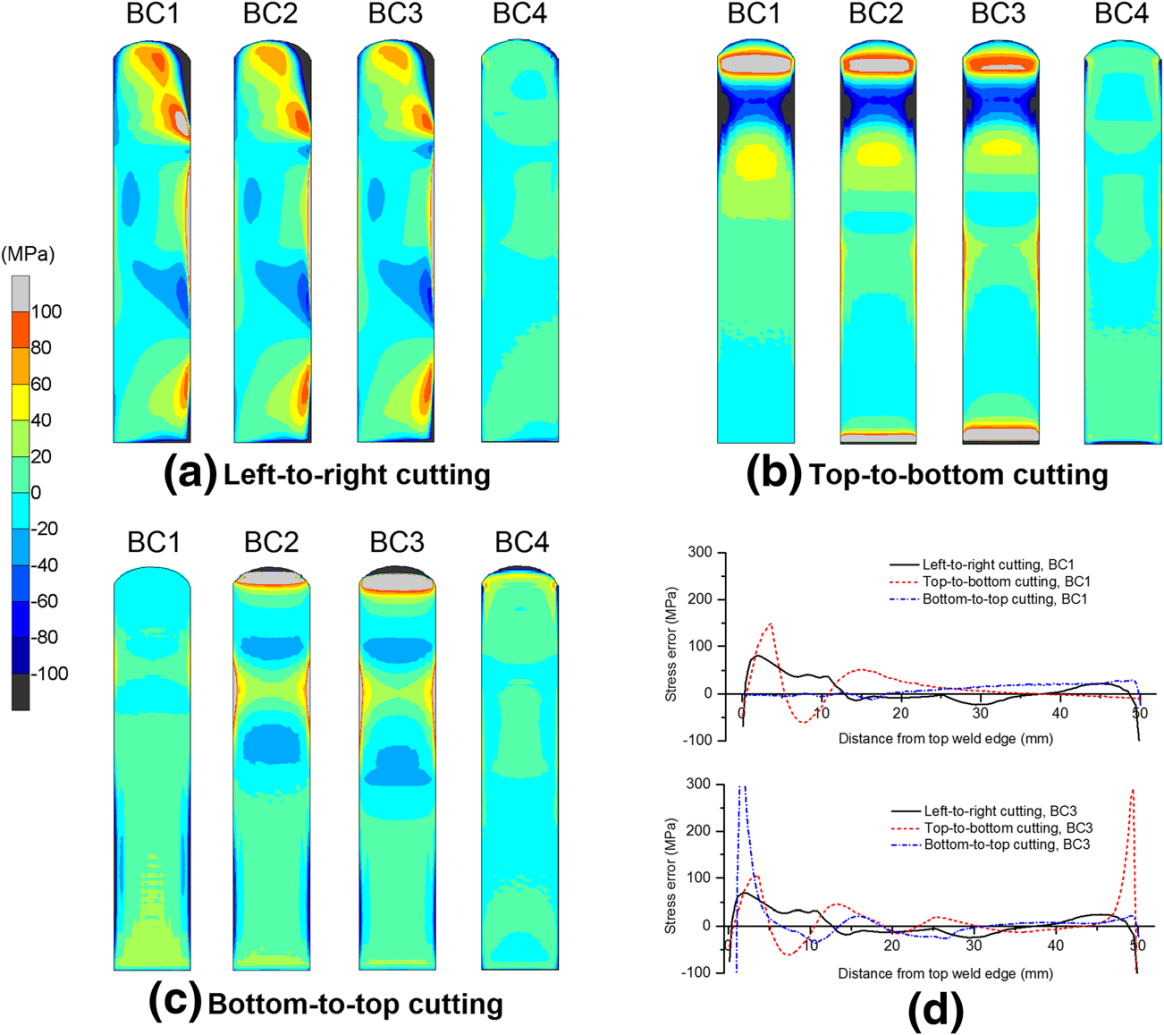
Stress errors calculated with respect to elastic cutting. Contour maps are shown in (a)–(c), and line profiles at the mid-width position of the sample are shown in (d)

For the beam examined here, the surface displacements clearly differ from those assumed to be produced by purely elastic relaxation during contour cutting, and the magnitudes of the deviations depend on the cutting strategy. Consequently, the errors in the back-calculated stresses, with reference to elastic cutting (or the true residual stress reconstructed), vary for different cutting strategies (see Fig. 11). These displacement deviations and stress errors are caused by cutting-induced plasticity (Figs. 7 and 8) as a result of stress redistribution after the relief of the stresses in the material that was removed. However, if the stresses within the removed material are self-equilibrating themselves, no plasticity can be induced since the cutting will not cause any redistribution of stresses. For instance, Kim et al. [30, 31] simulated the cutting of a four-point bend prismatic bar and a thin plate, which had constant residual stresses through the thickness; they found that the cutting in through-thickness direction caused negligible plastic deformation, which was attributed to the self-equilibrating stress state within the material that was removed. Unfortunately, in many cases, stress redistribution occurs during cutting.

Vertical cutting of the edge-welded beam preserves the symmetry inherent in the weld residual stress distribution, whereas horizontal cutting produces asymmetrical stress fields, as shown in Fig. 9. Cutting-induced plasticity can lead to artefacts in the stress distribution, e.g. drops in stress at sample edges (see Figs. 9 and 10). From the back-calculated stress maps in Fig. 9, it is evident that, overall the form of the stress distribution is skewed towards the end of the cut in most cases; and the most significant errors in stress are generally produced in the regions where plastic strain is concentrated (see Figs. 7, 8, 9, 10 and 11). Regarding the difference between least-constraint and clamping for vertical cutting, the former likely produces large stress errors at the start-of-cut stage while the latter produces large errors at the end-of-cut stage. This implies that to avoid large errors, in the least-constraint case the cut should end in the region of major interest for residual stress measurement, contrary to the clamping case.

In experimental practice, it has been recommended that stress calculations at edges should not be reported, since the measured edge contour is usually unreliable [4, 11]. The present cutting simulations show that cutting-induced plasticity can also introduce significant errors near the sample edges. Therefore, the stress results at these locations should be used with caution due to potential plasticity issues, even when other edge-related errors have been eliminated.

From a statistical point of view, cutting-induced plasticity constitutes a bias error that is dependent on the stress state and the cutting strategy, and these may interact with other stochastic sources of error associated with measurements and data processing. Experimentally, the measured and averaged surface contours are usually smoothed and fitted to continuous functional forms for later FE meshing and nodal displacement definition [5, 8, 9]. The smoothing and fitting are supposed to reduce stochastic errors. However, improvements in experimental measurements and data processing may not lead to more accurate stress determinations if the errors mainly arise from cutting-induced plasticity. Olson et al. [32] have proposed a method to estimate the uncertainties in stress caused by the contour measurement noise and fitting errors. However, in their study, the displacement errors were considered stochastically, neglecting the effects of the plasticity-induced displacement errors. For a more accurate evaluation of errors in future work, the combined effects of cutting-induced plasticity and other sources of error should be examined. Furthermore, when cutting-induced plasticity occurs, the errors inherent in contour results discourage “perfect” measurement and fitting which require much more effort but do not essentially improve the accuracy of the obtained stresses. This highlights the importance of the mitigation of cutting-induced plasticity, which potentially occurs in the first step of the contour method.

Mitigation of cutting-induced plasticity and thus reducing stress errors can be accomplished by enhancing the efficacy of clamping strategies (Figs. 7 and 11), which is the most straightforward approach but it may be difficult to implement in some practical cases. For instance, rigid clamping of the sample surfaces immediately adjacent to the cut plane can almost eliminate the plasticity associated with cutting, which has been achieved in the numerical simulation (in the case of BC4), but it requires sophisticated fixtures/tools and WEDM setup in an experiment. Therefore, the exploration of novel constraint methods is worthwhile. As mentioned in “Introduction” section, Traore et al. [14] used an embedded cutting configuration to impose self-constraint during sample cutting, providing an effective way to reduce plasticity-induced errors in contour method measurements. This approach was recently further improved by employing a double-embedded cutting configuration [18, 19]. However, the self-constraint approach requires production of pilot holes and several runs of the WEDM to complete the cutting, rather than the simple single cut normally implemented, and thus these novel self-constraint approaches may increase operational/processing complexity and introduce new errors due to the presence of pilot holes [18, 19]. As demonstrated through this study, optimising the cutting direction is also helpful in reducing plasticity, and this may be much easier to implement than the optimisation of constraint. For the beam examined here, a bottom-to-top cutting direction, i.e. from the base material to the weld region in a direction that falls within the plane of symmetry for the residual stress distribution, leads to minimum plasticity-induced errors in stress, when the least constraint condition (BC1) is applied, as shown in Fig. 11.

**Fig. 12:**
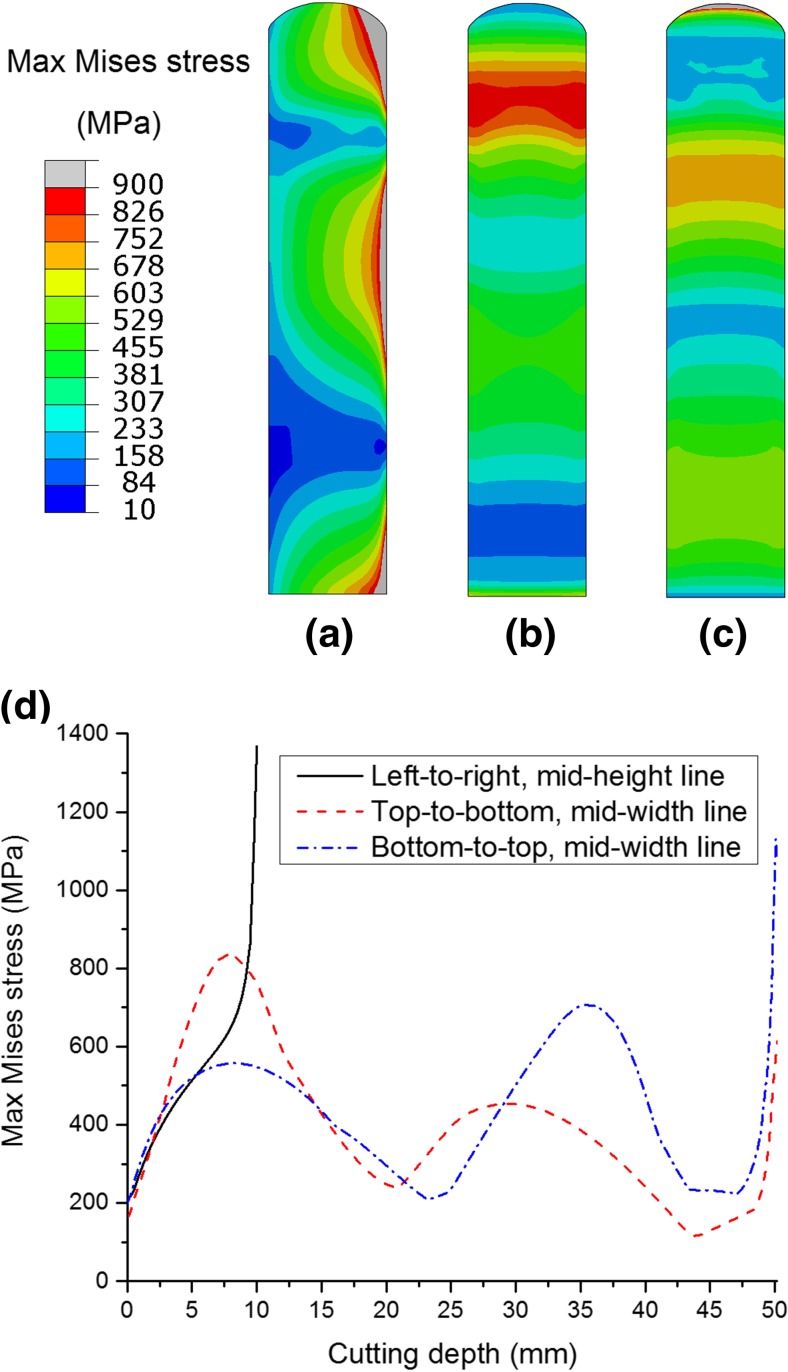
Maximum von Mises stress experienced on the cut surface in elastic cutting (BC2). Contour maps are shown for (**a**) left-to-right cutting, (**b**) top-to-bottom cutting and (**c**) bottom-to-top cutting; and line profiles for maximum von Mises stress (d)

Recalling the weld residual stress distributions in the beam, see Figs. 3 and 5(a), the magnitudes of the stresses are close to both the initial yield stress of the beam material (216.5 MPa at room temperature, defined in the combined hardening model, see Table 1) and its isotropically hardened value (e.g. 234.7 MPa at ${\overline{\varepsilon}}^{\mathrm{pl}}=0.05$ according to equation (5)). Thus it is unsurprising that cutting will induce significant plastic deformation for the case of inadequate clamping. Figure 12 shows the maximum von Mises stresses experienced on the cut surface during elastic cutting, under condition BC2, for the weld residual stress distribution under consideration. It can be seen that the von Mises stresses that are generated during cutting are much higher than the yield stress. Thus, plasticity effects could still be considerable, even when the magnitude of residual stresses is lowered (e.g. to one third of the yield stress), indicating the potential for plasticity-induced errors and the necessity of mitigating plasticity even when relatively low residual stresses are expected.

**Fig. 13:**
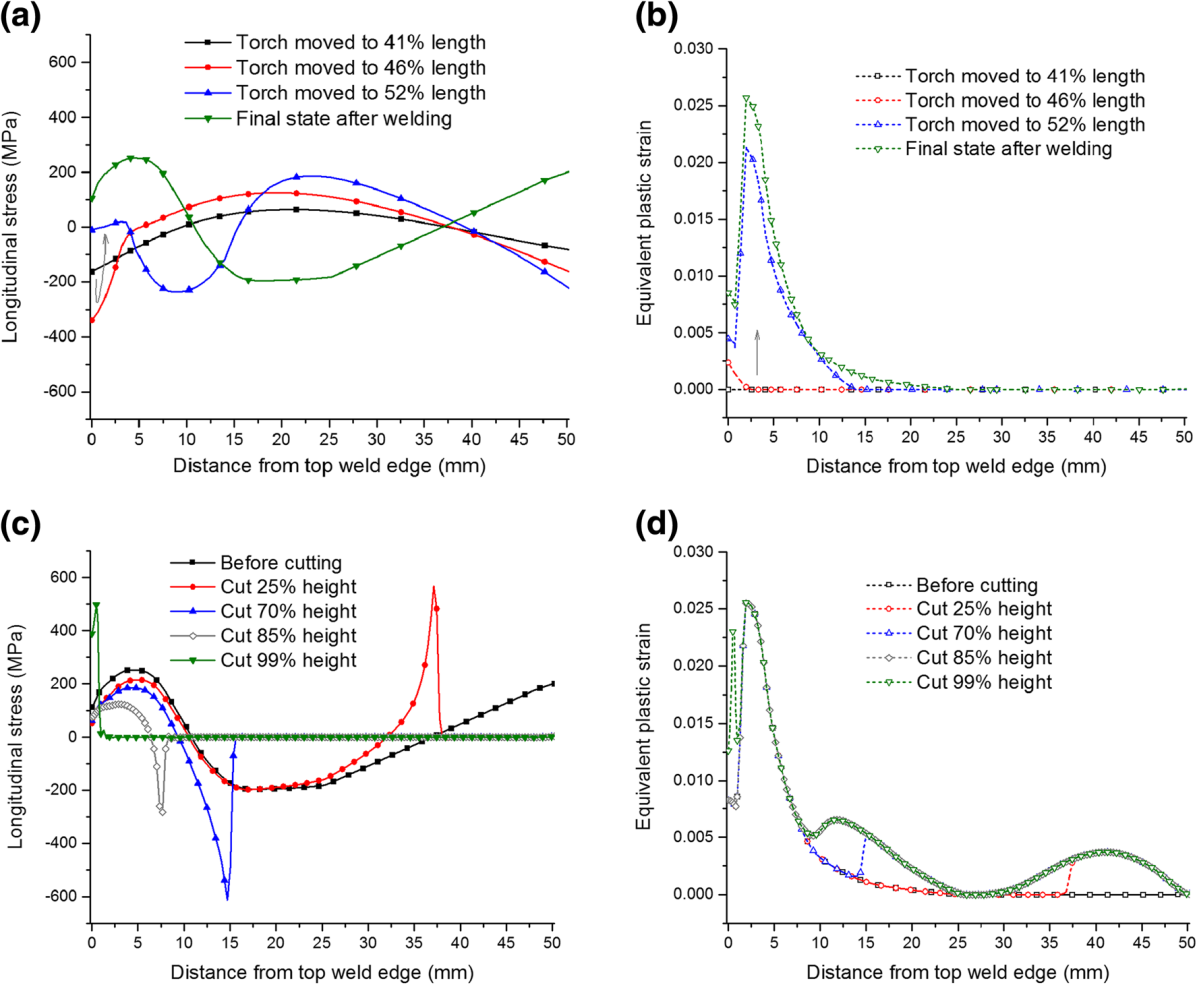
Longitudinal stresses (**a**) and equivalent plastic strains (**b**) when the welding torch (i.e. heat source) moved to different positions. Longitudinal stresses (**c**) and accumulated equivalent plastic strains (**d**) when the cutting proceeded from bottom to top (BC2). The distributions are evaluated along a line at the mid-width position on the mid-length plane

Cutting-induced plasticity can also be affected by the cyclic strain hardening behaviour if the material experiences cyclic yielding, as the Bauschinger effect will reduce the yield stress when reverse yielding occurs (see Fig. 2), thereby increasing the propensity for plastic deformation. Figure 13(a) and (b) show the histories of the longitudinal stress and equivalent plastic strain at the mid-width position on the mid-length plane during welding. It is evident that after welding, most of the plastic strain is located in the top half of the beam, while the bottom half remains elastic. It can be also clearly seen that the majority of the plastic deformation developed when the heat source moved from a position that was 46% of the way along the length to 52%, during which the longitudinal stress was mainly compressive. Afterwards, for material within a distance of ~8 mm (~16% of the sample height) from the top edge of the weld, reverse yielding occurred under tension. Figure 13(c) and (d) show the histories of the longitudinal stress and accumulated equivalent plastic strain at the mid-width position on the mid-length plane during bottom-to-top cutting (BC2). When the tip of the cut moved from the bottom end towards the mid-height plane, it was subjected to tensile stress, and the material, which had previously deformed elastically during welding, yielded. The material located in a region between ~7.5 and ~10 mm away from the top edge of the weld, corresponding to a span equal to ~5% of the sample height, experienced moderate reverse yielding (second reversal with reference to the original material state before welding), while at some other locations, plastic deformation increased, with a flow direction similar to that during welding.

**Fig. 14:**
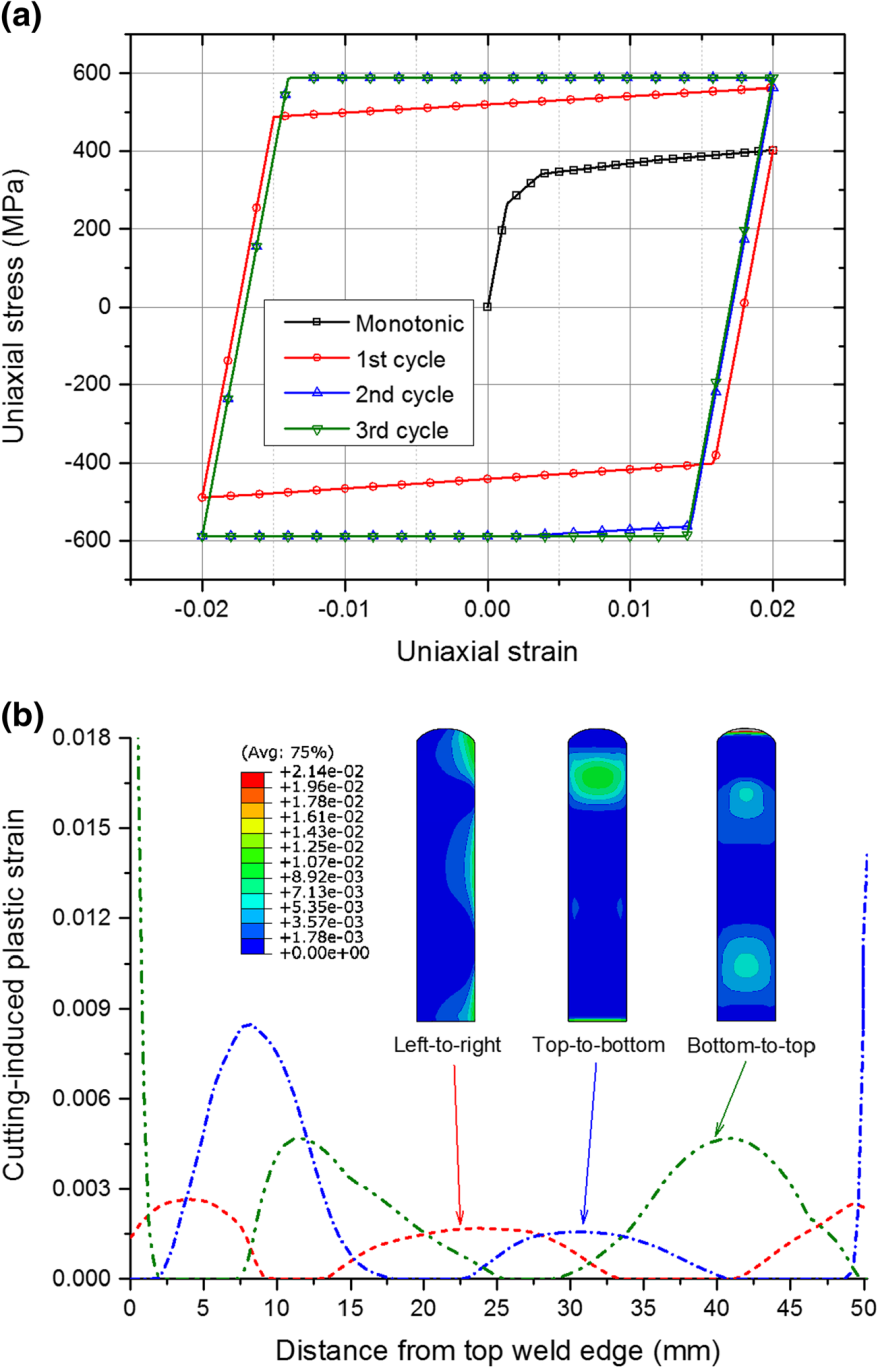
(**a**) Uniaxial cyclic stress-strain curves predicted by an isotropic hardening model; (**b**) line profile comparisons of the cutting-induced equivalent plastic strains at the mid-width position on the cut surface for different cutting directions under BC2, when the isotropic hardening model is used in both welding and cutting simulations (inset shows the contour maps for the cutting-induced plasticity)

Additional simulations of welding and cutting have been performed using an isotropic hardening material model, as shown in Fig. 14(a), in order to evaluate the potential effect of the cyclic strain hardening behaviour on the cutting-induced plasticity. Comparing Fig. 14(b) with Figs. 7 and 8(b), we can see that the cutting-induced equivalent plastic strain is overall insensitive to the hardening model adopted for the edge-welded beam. This is primarily because only moderate reverse yielding occurs in a narrow region, during cutting, as shown in Fig. 13(c) and (d). Despite the overall similarity it is discernible that, for vertical cutting, the cutting-induced plastic strain associated with the isotropic hardening model is actually slightly larger than that associated with the combined hardening model. This can be attributed to the overestimation of weld residual stresses using an isotropic hardening model [25, 27]. However, the effects of cyclic strain hardening may become more pronounced if extensive and significant reverse yielding occurs during cutting, but this is a topic for future work.

## Conclusions

The entire contour method process has been simulated for a benchmark edge-welded beam sample to numerically evaluate the errors associated with cutting-induced plasticity in residual stress measurements. Welding and cutting models were both validated through comparisons with previous experimental results.

The cutting simulations showed that the extent of plastic deformation and the resulting errors in stress are dependent on both the cutting directions and the boundary conditions that are selected to represent the clamping configuration. For the beam that was examined, the symmetry inherent in the residual stress distribution, about the mid-width plane, was violated when the cutting direction did not fall within the plane of symmetry, i.e. when horizontal cutting (left-to-right) was employed. The back-calculated stresses for the different clamping conditions that were considered in this study exhibited the following features: (1) overall, the form of the stress distribution was skewed towards the end of the cut; (2) significant errors in stress were produced in the regions where plastic deformation was concentrated; (3) the edges of the sample were most susceptible to errors in stress, particularly for the end-of-cut location where large plastic strains were produced; and (4) errors in stress were almost eliminated and became insensitive to the cutting direction when adequate clamping (immediately adjacent to the plane of cut) was imposed. For the least-constraint case (i.e. solely preventing rigid body motion), the stress errors were mainly produced at the start-of-cut stage for vertical cutting and this implies that the start of the cut should not be located in a region of major interest. More importantly, minimum errors in stress were achieved through bottom-to-top cutting in the direction (within the residual stress symmetry plane) from the base material to the weld region.

The conclusions summarised above are valid for the edge-welded beam, as well as those materials or structures having similar residual stress and yielding behavior. More investigations may be needed for other types of residual stress distributions and their associated cyclic yielding. Most importantly, this study demonstrated that it is necessary to record and use cutting process information for estimating the potential for plasticity-induced errors associated with contour method results, and that the cutting direction and clamping strategy can be optimised with guidance from numerical models.
